# Rapid and robust derivation of mesenchymal stem cells from human pluripotent stem cells via temporal induction of neuralized ectoderm

**DOI:** 10.1186/s13578-022-00753-2

**Published:** 2022-03-15

**Authors:** Wei Jin, Yi He, Tuo Li, Fei Long, Xin Qin, Yuan Yuan, Ge Gao, Hosen Md Shakhawat, Xinguang Liu, Guoxiang Jin, Zhongjun Zhou

**Affiliations:** 1 School of Biomedical Sciences, LKS Faculty of Medicine, The University of Hong Kong, Hong Kong, China; 2 Chinese Academy of Sciences Regenerative Medicine of Hong Kong, Hong Kong, China; 3 Department of Endocrinology, Chang Zheng Hospital, Shanghai, 200003 China; 4 Guangdong Provincial Key Laboratory of Medical Molecular Diagnostics, Institute for Aging Research, Guangdong Medical University, Dongguan, China; 5 Medical Research Center, Guangdong Provincial People’s Hospital, Guangzhou, China; 6 Shenzhen Hospital, The University of Hong Kong, Shenzhen, China

**Keywords:** ESCs, iPSCs, MSCs differentiation, HGPS, Aging, Neural ectoderm

## Abstract

**Background:**

Mesenchymal stem cells (MSCs) are emerging as the mainstay of regenerative medicine because of their ability to differentiate into multiple cell lineages. The infinite proliferative potential of human pluripotent stem cells (PSCs) grants an unlimited supply of MSCs. Despite their great potential in therapeutic applications, several drawbacks have hindered its clinical translation, including limited number of replication, compromised potential and altered function in late passages. The aim of this study is to establish an efficient method for the production of MSCs from pluripotent stem cells for potential clinical application in rare human disease Hutchinson-Gilford progeria syndrome.

**Results:**

We established a robust method allowing rapid derivation of MSCs from both human iPSCs and ESCs via a temporal induction of neural ectoderm in chemically defined media. The iPSC- and ESC-derived MSCs satisfy the standard criteria of surface markers. They exhibited a high tri-lineage differentiation potential with over 90% transcriptional similarity to the primary MSCs derived from bone marrow. To evaluate the potential application of this method in disease modeling, MSCs were generated from iPSCs derived from a patient with Hutchinson-Gilford progeria syndrome (HGPS-MSCs) and from mutation-rectified HGPS-iPSCs (cHGPS-MSCs). HGPS-MSCs manifested accelerated senescence whereas mutation rectification rescued cellular senescence in HGPS-MSCs.

**Conclusions:**

The robust method of MSC derivation from ESCs and iPSCs provides an efficient approach to rapidly generate sufficient MSCs for in vitro disease modeling and clinical applications.

**Supplementary Information:**

The online version contains supplementary material available at 10.1186/s13578-022-00753-2.

## Introduction

Mesenchymal stem cells (MSCs) are multipotent progenitor cells firstly isolated from bone marrow [1]. MSCs are characterized by their fibroblast-like morphology, high potential of osteogenesis, adipogenesis and chondrogenesis, positive for a panel of typical surface markers, including CD73, CD90 and CD105 [2]. Because of the low immunogenicity, high regenerative capability and strong immunomodulatory properties, both allogenic and autologous MSCs have been deployed in a large number of phase I-III clinical trials for degenerative diseases and autoimmune disorders, such as ischemic heart diseases, neurodegenerative disorders, graft versus host disease and Crohn’s disease, as well as hematopoietic stem cell (HSC) engraftment failure [3, 4]. Both ex vivo and animal studies have shown that MSCs can home to sites of the injuries or inflammation to create a favorable microenvironment to support the survival and functional recovery of injured cells, promoting their proliferation via direct cell–cell contact or paracrine secretion [5]. In addition, MSCs in culture secrete a myriad of bioactive molecules, including growth factors, cytokines, chemokines, and mRNA/microRNA-containing macrovesicles [6, 7], which have been successfully used in clinical cosmetology [8]. Therefore, MSCs have emerged as the most frequently used bio-reagent in the fields of regenerative medicine and tissue engineering [9].

MSCs have been successfully isolated and expanded from multiple human tissues including bone marrow, adipose tissue, umbilical cord, muscle, liver, cartilage, and lung [10, 11]. However, there are several drawbacks in MSCs derived from adult tissues. The quantity of MSCs isolated from primary tissues is far from sufficient for clinical applications [12] and the primary MSCs usually exhibit limited proliferative capacity, altered functionality and compromised differentiation potential over long-term culture [13]. In addition, tissue-derived MSCs suffer from tissue and donor variability, especially with the regard of age and disease-status of the donors [14, 15], which may explain the discrepancy in the efficacies of preclinical evaluation of MSCs. On the other hand, although MSCs exhibit low immunogenicity in general, the allogenic MSCs from bone marrow (BM-MSCs) were reported to induce undesired immune response in several animal models [16–18].

Human pluripotent stem cells (hPSCs), including embryonic stem cells and induced pluripotent stem cells, possess the capacity of indefinite proliferation and differentiation into all three germ layers, making hPSCs an ideal, easily accessible and safe source for large-scale production of high-quality MSCs. Up till now, various strategies and modified protocols have been developed to generate MSCs from hPSCs. Barberi et al. co-cultured hESCs with mouse OP9 cells for 40 days followed by FACS sorting for CD73 + cells to enrich the MSC population [19]. MSCs can be derived and purified with typical surfaces markers from the spontaneous differentiation of embryonic body (EB), a sphere culture derivatives of hPSCs. The EB-mediated derivation of MSCs has been extensively optimized to improve the yields, for example, plating EB to gelatin coated-dishes and expanding monolayer cells before sorting, or continually splitting the cells until a homogeneous fibroblastic morphology appears. Lian and colleagues cultured hESCs directly for 7 days in MSCs medium supplemented with PDGF-AB and bFGF without EB formation followed by sorting for CD105^+^/CD24^−^ cells [20]. The disadvantages of these methods are time-consuming and inefficient. In addition, the use of animal components makes the MSC production non-compliant with the clinical requirements. To establish a direct induction platform, Sánchez et al. blocked the TGF-beta signaling with small molecule inhibitor SB431542. This could promote the induction of MSCs from hESCs, but not iPSCs [21]. Based on this method, Zhao et al. prolonged the induction in the presence of SB431542 and 7.5% CO_2_ to enable MSCs derivation from iPSCs [22]. Later, Wang et al. generated MSCs via transforming hESCs into a trophoblast-like stage before differentiating to MSCs [23], which may give rise to ethical controversies in clinical applications of hESCs. Neural crest cells (NCCs) belong to a transitional multi-potent population emerged during development of embryonic ectoderm in vertebrates, which can be easily directed towards mesenchymal derivatives [24]. Fukuta et al. previously described the 7-days induction of hPSCs to neural ectoderm with TGF-beta inhibition and WNT activation followed by FACS sorting for p75^high^ population of NCCs before MSCs induction [25]. However, the efficiency of NCC production is relatively low and FACS sorting is not practical for large-scale production of MSCs. In addition, highly differentiated pure NCCs requires specific medium with sophisticated composition and approximately 20 days induction, adding extra burden for MSCs production [26].

Here, we report a robust method to derive MSCs with high efficiency from both hESCs and iPSCs through a simple two-stage culture. To minimize potential undesired effects in human iPSCs generation, an integration-free minicircle vector was employed for the reprogramming of dermal fibroblasts. For MSCs differentiation, hPSCs were firstly induced to neural ectoderm in chemically defined-medium for 5–7 days. Upon switching to MSC medium, fibroblast-like cells appeared as early as day 4 and became homogenous at passage 3. Flow cytometry analyses showed that over 95% of hPSCs-derived MSCs were CD73 + /CD90 + /CD44 + and less than 1% cell expressed hematopoietic markers (CD11b/CD34/CD19/CD45/HLA-DR). hPSCs -derived MSCs exhibited high potential in differentiation into osteo-, adipo- and chondro-lineages.

Hutchinson-Gilford progeria syndrome (HGPS) is a rare premature aging syndrome, caused by a dominant mutation in *LMNA* gene. To test if hPSC-derived MSCs could serve as a disease model, we induced the MSCs from iPSCs derived from a patient with HGPS. HGPS-MSCs recapitulated the accelerated cellular senescence, including nuclear blabbing, attenuated DNA damage repair kinetics and abnormal epigenetic modifications. When the mutation was rectified in HGPS-iPSCs by a CRISPR/CAS9-based double selection strategy, mutation corrected MSCs were rejuvenated both in vitro and in vivo, providing a feasible tool for stem-cell based intervention in HGPS.

## Results

### Generation and characterization of human iPSC via integration-free minicircle vector

**Fig. 1:**
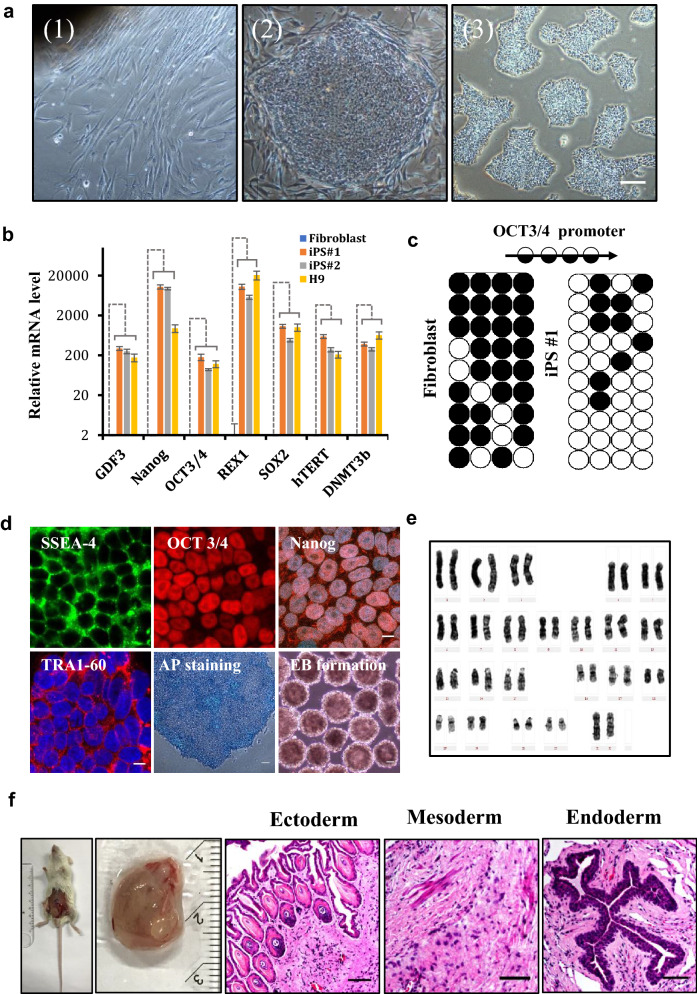
Generation and characterization of human iPSCs with an integration-free minicircle vector. **a** Morphology of human primary dermal fibroblasts (1), iPSCs generated from fibroblasts cultured on feeder layer (2) and feeder-free plate (3). Scar bar 100 μm. **b** Expression of pluripotency-related genes *OCT3/4,* NANOG, *SOX2, REX1, GDF3, hTERT* and *DNMT3b*, analyzed by qPCR. Gene expression was normalized to endogenous *GAPDH* and was plotted against fibroblast, H9 served as a positive control. Data represent mean ± S.D. n = 3. **c** DNA methylation detected by Bisulfite sequencing in the genomic region of *OCT3/4* promoter in iPSCs and its parental dermal fibroblasts. Open and filled circles represents unmethylated and methylated CpGs, respectively. Each row represents bisulfite sequencing result from a given amplicon and ten amplicons were analyzed. **d** Immunostaining of pluripotency markers SSEA-4, TRA60-1, NANOG and OCT3/4 (Scar bar 10 μm), Alkaline Phosphatase (AP) staining and embryoid body (EB) formation of iPSCs. Scar bar 100 μm. **e** Karyotyping assay of iPSC with a normal diploid female karyotype. **f** Teratoma formation of iPSCs in the testis of NOD/SCID mice. Teratoma and H&E staining of paraffin sections to reveal three germ layers. Scar bar 100 μm

Although somatic cell nuclear transfer (SCNT) mediated generation of ESCs and animal cloning have achieved remarkable progress in animals and human, ethical issues remain as an obstacle in clinical applications. Patient-specific iPSCs have similar potential as ESCs for disease modeling, drug screening and regenerative medicine without much ethical concerns, providing a better source for personalized stem cell applications. However, majority of the strategies in iPSC derivation are based on viral integration which may lead to oncogenic mutations. To address the safety concerns, we employed a minicircle vector reported previously to generate human iPSC from primary cells (Additional file 1: Fig. S1a). Dermal skin from a healthy donor was used for fibroblast preparation. To minimize the impact of cellular senescence on reprogramming [27], we started the nucleofection with MIP 247 vector in fibroblasts within passage 10. The process of reprogramming is illustrated in (Additional file 1: Fig. S1a**)**. At least six independent iPSCs clones were collected and expanded on either autologous feeder-layer or Matrigel (Fig. 1a). Alternatively, we also used renal epithelial cells collected from urine to derive iPSCs to avoid the invasive approach in biopsy **(**Additional file 1: Fig. S1c**)**.

To characterize these iPSCs, genomic DNA were extracted to confirm the absence of minicircle plasmid insertion with four pairs of primers (Additional file 1: Fig. S1b). As shown in **(**Additional file 1: Fig. S1d**)**, neither iPSC clones nor the H9 hESCs were positive for vector insertion. The iPSCs expressed pluripotency markers, such as *GDF3, OCT3/4*, NANOG, *REX1, SOX2,* and *DNMT3b*, at levels comparable to that in human ESC H9 **(**Fig. 1b). Bisulfite sequencing at *OCT3/4* locus in the genomic DNA revealed a significant reduction in DNA methylation in iPSCs (Fig. 1c), indicating epigenetic memory has been erased during reprogramming. The pluripotency was also confirmed by immunostaining of SSEA4, OCT3/4, NANOG, TRA1-60, and Alkaline Phosphatase (AP) activities, as well as the capability of in vitro embryoid body (EB) formation (Fig. 1d). Genome integrity was shown by normal chromosome pairing (44 + XX) in karyotyping examination (Fig. 1e). In addition, when injected into immunocompromised mice, these iPSCs gave rise to three germ layers demonstrated by the hematoxylin and eosin (H&E) staining in the teratoma formation assay (Fig. 1f). Taken together, iPSCs generated from human primary cells are footprint free and clinically compliant, given that no animal components were used in the primary cell culture and reprogramming process.

### Generation of Mesenchymal stem cells from human PSCs by a two-stage induction approach

MSCs can be induced from hPSCs through an intermediate stage, the NCCs [24–26, 28–30]. However, the efficiency of NCCs induction from hPSCs is relatively low. The use of flow cytometric selection for HNK1/p75^++^ to purify NCCs is not practical for large-scale production of MSCs with a possibility of introducing undesired contamination [25, 31]. Recently, Menendez et al. reported that over 85% pure NCCs can be generated directly from hPSCs without FACS sorting. However, the in-house made culture media for hPSCs and for NCCs induction are complicated. The entire process of NCCs induction takes more than 20 days [26] and two extra weeks are then required for transition from NCCs to MSCs. Given that 1) NCCs from neural ectoderm are easily transdifferentiated into MSCs with high efficiency; 2) non-NCC cells derived from neural ectoderm, such as neurons and astrocytes, are more hypersensitive to stress than MSCs, it is plausible that MSCs can be directly and rapidly induced from neural ectoderm cells without NCC purification.

**Fig. 2:**
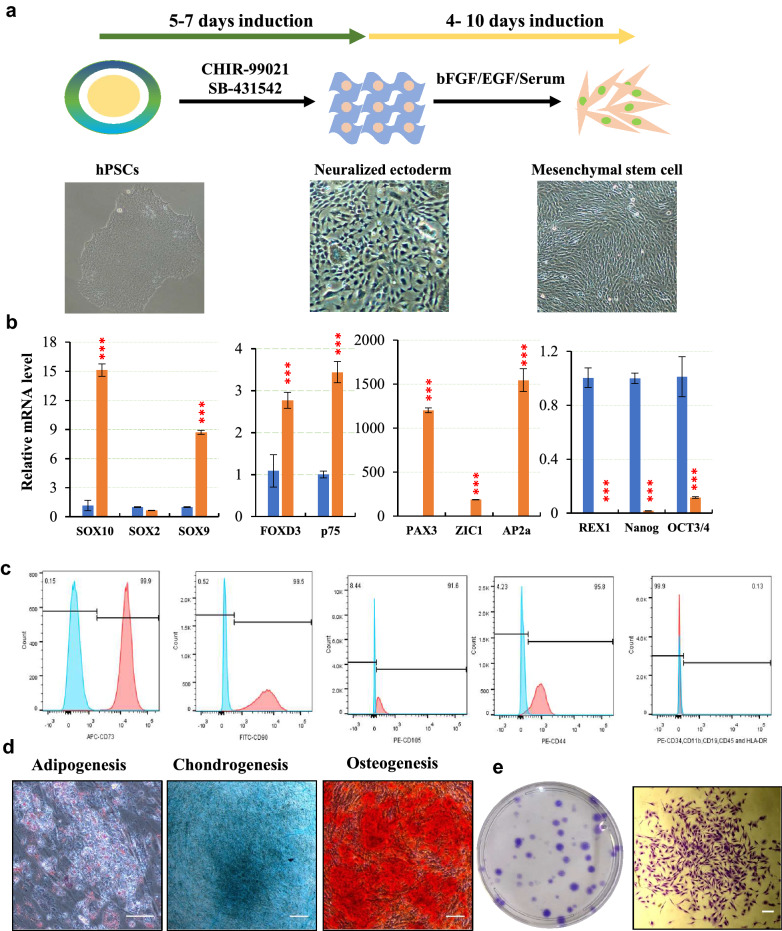
Generation of MSCs from pluripotent stem cells by two-step induction via neuralized ectoderm as a differentiation intermediate. **a** Schematic illustration of two-step procedure for MSCs induction. Human pluripotent stem cells are treated with SMAD inhibitor and WNT activator for 5–7 days to differentiate to the neural ectoderm. Cells are then split before further incubated with medium supplemented with bFGF/EGF. Fibroblast-like cell morphology were observed around day 4–10. Cells were expanded for another 2–3 passages followed by FACS analysis of typical MSC surface markers. Lower panel shows the morphology of initial human iPSCs, intermediate neural ectoderm and fibroblast-like MSCs. Scar bar 100 μm. **b** Expression of neural ectoderm genes (*SOX10, SOX2, SOX9, FOXD3, p75, PAX3, ZIC,* and *AP2a*) and pluripotency related genes (*OCT3/4*, *NANOG*, and *REX1*) examined by qPCR. Gene expression was normalized to endogenous *GAPDH*. Relative mRNA levels were plotted against that in iPSCs. Data represent mean ± S.D. n = 3; *p < 0.05, ** *p* < 0.01, *** *p* < 0.001, **** *p* < 0.0001, ns, not significant; one-way ANOVA coupled with *Tukey’s *post hoc test was used for statistical analysis. **c** Flow cytometry analyses of typical positive (CD73, CD90, CD105 and CD44) and negative (CD45, CD34, CD11b, CD19 and HDL-DR) MSCs surface markers. **d** MSCs are induced toward adipogenesis, chondrogenesis and osteogenesis in vitro for three weeks. Adipocyte, chondrocyte, and osteoblast are detected by staining of Oil red O, Alizarin red S and Alcian blue. Scar bar 100 μm. **e** Colony formation of MSCs. MSCs are stained with crystal violet. A representative colony in high magnification is shown on the right

To verify the hypothesis, we firstly induced hPSCs to neural ectoderm using modified medium composed of 48.5% of Neurobasal medium, 48.5% of DMEM/F12 basal medium, 1% of N_2_ and 2% of B27, 10 μM of SB431542 (TGF-β pathway inhibitor) and 3 μM of ChIR99021 (WNT signal activator). After induction for 5 days, cells exhibited neural-like morphology, spreading out and separating from each other, though small patches of hPSCs-like clusters may still be observed (Fig. 2a). The qPCR analysis revealed that *OCT3/4*, *NANOG*, and *REX1* were dramatically decreased while neural ectoderm-associated genes including *PAX3*, *ZIC1*, *SOX10*, *SOX9*, *AP2a*, *FOXD3* and *p75*, were upregulated (Fig. 2b). In line with this, immunostaining showed loss of pluripotent markers OCT3/4, NANOG and AP2α and expression of PAX7 and typical neural progenitor/stem cell marker NESTIN (Additional file 1: Fig. S2c). The induced neural ectoderm cells were capable of suspension culture (Additional file 1: Fig. S2a). p75 has been identified as one of the MSCs markers in vivo*.* [32] Ninety-nine percent of neural ectoderm cells were found positive for p75 (Additional file 1: Fig. S2b). However, the percentage of p75^high^ was lower compared with previous study. [26] Upon culture for two weeks, these neural ectoderm cells were able to be differentiated into adipocytes, chondrocytes, and osteocytes in corresponding induction media (Additional file 1: Fig. S2d). Collectively, these data suggest that these neural ectoderm cells are not pure NCCs, rather populations manifesting MSCs-like properties.

To induce MSCs from neural ectoderm cells, we switched the culture medium directly to MSC medium containing 10% of serum supplemented with 2 ng/ml of bFGF and 2 ng/ml of EGF. To generate clinical compliant MSCs, human umbilical cord blood serum (hUCBS) was used in the medium instead of FBS during the induction of MSCs. Spindle-like morphology was initially observed on day 4 after medium switch. Cells were split at a ratio 1:1 or 1:2 when reached full confluency. Moderate cell death was observed at the first two passages. After three passages, the cells became homogenous with enhanced proliferation rate. Flow cytometry analyses showed over 90% of the cells were positive for typical MSC surface markers (CD73 99.9%, CD90 99.5%, CD105 91.6% and CD44 95.8%) and negative for CD45, CD34, CD11b, CD19 and HDL-DR (< 1%) **(**Fig. 2c). To examine if these MSCs retain full differentiation potentials, MSCs at passage 5 were induced for adipogenesis, chondrogenesis and osteogenesis. As shown in Fig. 2e, differentiated cells were stained positively by Oil red O, Alcian blue, and Alizarin red (Fig. 2d). Colony formation assay showed a strong self-renewal capacity of the neural ectoderm cells-derived MSCs (Fig. 2e).

### hPSCs-derived MSCs exhibit similar properties and gene expression profile as bone marrow-derived MSCs

**Fig. 3:**
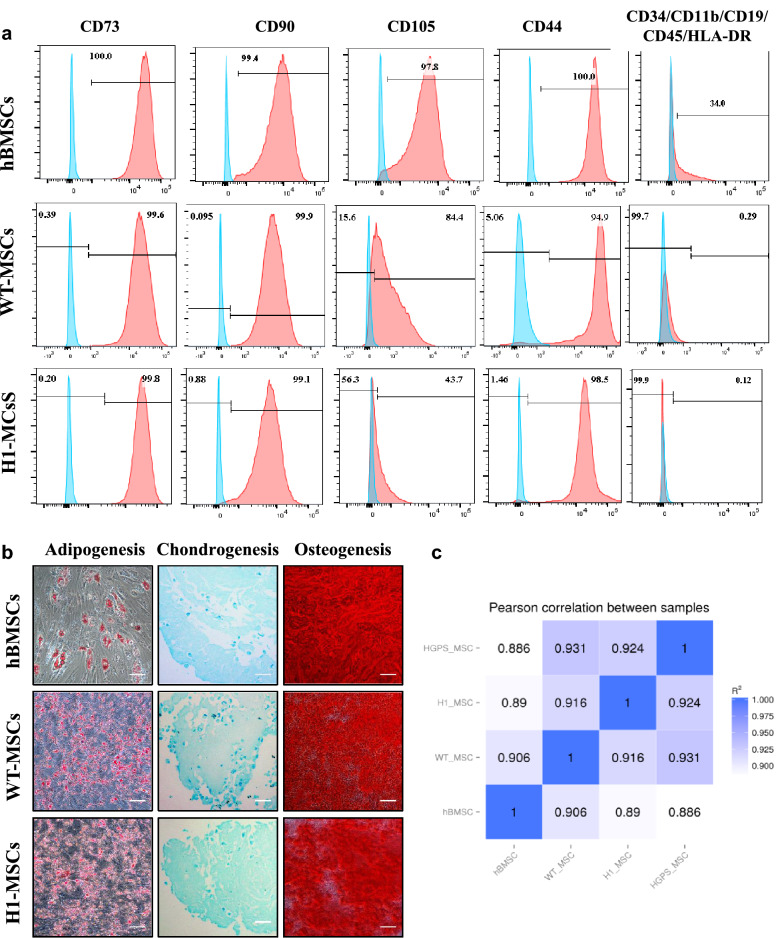
Comparison of iPSCs- and ESCs-derived MSCs with primary bone marrow MSCs. **a** Flow cytometry analyses of typical positive (CD73, CD90, CD105 and CD44) and negative (CD45, CD34, CD11b, CD19 and HDL-DR) surface markers for MSCs in WT-MSCs, H1-MSCs and BM-MSCs. **b** Adipogenesis, chondrogenesis and osteogenesis of WT-MSCs, H1-MSCs and BM-MSCs. Adipocyte, chondrocyte, and osteoblast are detected by the staining of Oil red O, Alizarin red S and Alcian blue, respectively. Scar bar 100 μm. **c** Correlation co-efficient (R2) of gene expression profiling between WT-MSCs, H1-MSCs and BM-MSCs

Differences have been reported in the efficiency of MSC induction between human ESCs and iPSC. It is in general easier for ESCs than iPSCs to differentiate into MSCs as the later require longer time and specific stress adaptation [21, 22]. To investigate whether the reduced efficiency of differentiation in iPSCs is a result of the clonal variation of iPSC quality, we derived MSCs from the second human iPSCs clone (WT-MSCs) and human ESCs H1 (H1-MSCs). Interestingly, the iPSCs and ESCs showed comparable differentiation efficiencies in our hands and similar MSCs surface markers including CD73, CD90, and CD44, with an exception for CD105 exhibiting higher percentage in iPSC-derived WT-MSCs (84%) than in H1-MSCs (43%) (Fig. 3a). The variable percentage of CD105 is in line with the previously reported NCCs-induced MSCs [33, 34]. Overall, these results demonstrated that MSCs could be robustly and efficiently generated within a substantially shorter time from both human ESCs and iPSCs by our protocol.

To explore the similarity and difference between hPSCs-derived MSCs and mature MSCs directly isolated from tissues, we took primary bone marrow MSCs (BM-MSCs) for comparison. BM-MSCs exhibited high percentage of surface markers CD73, CD90, CD105 and CD44. However, a sub-population of these BM-MSCs were also positive for CD34, CD11b, CD19, CD45 and HLA-DR cocktails (Fig. 3a). When differentiation capabilities were examined, adipocyte, chondrocytes and osteoblasts were all successfully induced by BM-MSC, ESCs-MSCs and iPSCs-MSCs verified by staining of Oil Red O, Alcian Blue and Alizarin Red S (Fig. 3b). Of note, the oil droplets in BM-MSCs was found significantly larger than that observed in H1-MSCs or WT-MSCs (Fig. 3b), which is consistent with the previous reports [22–24]. To further compare these MSCs generated from different sources, gene expression profiles were analyzed. As shown in Fig. 3c, a high correlation co-efficient (R2) was observed between BM-MSCs and either WT-MSCs (90.6%) or H1-MSCs (89.0%), suggesting that both hPSCs-derived MSCs and ESCs-derived MSCs are highly similar to primary BM-MSCs. The hPSCs-derived MSCs appeared to be more homogenous with 91.6% similarity (H1-MSCs vs WT-MSCs) (Fig. 3c). While BM-MSCs went cell cycle arrest after passage 5, hESCs derived MSCs did not show cellular senescence before passage 20.

### Generation and characterization of iPSCs from a HGPS patient

The human progeroid syndrome Hutchinson-Gilford progeria syndrome (HGPS) is predominantly caused by an autosomal dominant mutation (*p.G608G, c.1824C.* > *T*) in exon 11 of *LMNA* gene, resulting in the exposure of a cryptic splicing site and generation of a truncated mutant lamin A isoform, termed progerin. Progerin is an immature truncated lamin A missing 50 amino acids covering the second cleavage site. It is permanently farnesylated, therefore tightly attaches to the nuclear envelope, leading to the deformation of nuclear architecture, genome instability and epigenetic alternations [35]. Progerin expression is also observed in the elderlies [36] and ectopic expression of progerin in normal cells recapitulates accelerated cellular senescence in vitro and results in the collapse of tissue homeostasis in vivo. [37–39] Interestingly, neural cells of HGPS patients are unaffected, whereas mesoderm linages, especially mesenchymal cells, are severely affected [40–42], implicating HGPS-MSCs as an ideal cell model for stem cell aging and drug screening.

**Fig. 4:**
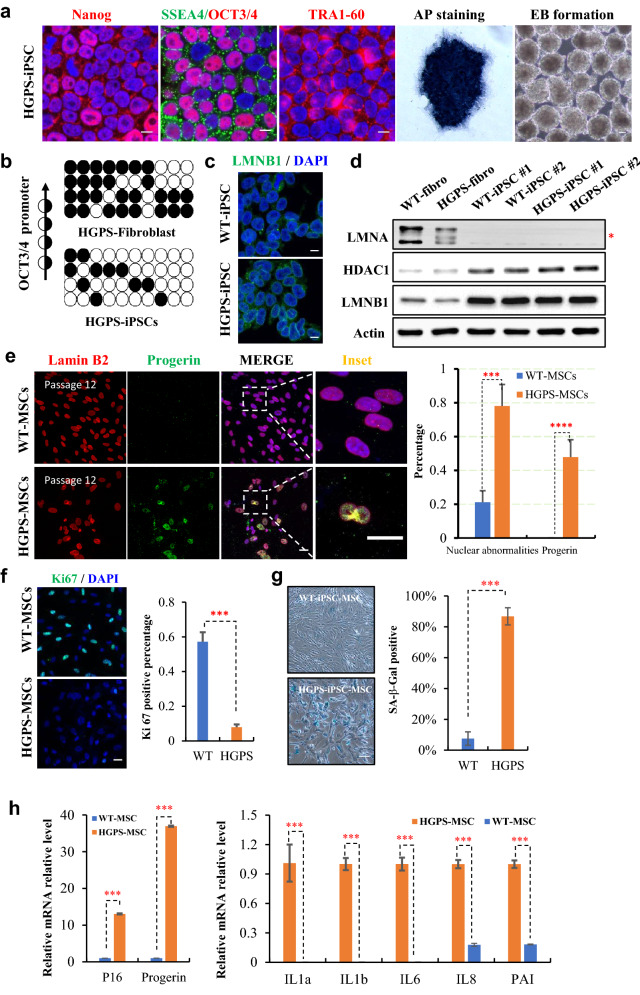
Derivation and characterization of HGPS-MSCs. **a** Immunostaining of SSEA-4, TRA60-1 and NANOG and OCT3/4 (Scar bar 10 μm), alkaline Phosphatase (AP) activity and embryoid body (EB) formation of HGPS-iPSCs. Scar bar 100 μm. **b** DNA methylation in the genomic region at *OCT3/4* promoter in HGPS-iPSCs and its parental dermal fibroblasts. Open and filled circles represent unmethylated and methylated CpGs, respectively. Each row represents bisulfite sequencing result from a given amplicon and ten amplicons were analyzed. **c** Immunostaining of lamin B1 in HGPS-iPSCs and WT-iPSCs. Scar bar 10 μm. **d** Expression of nuclear lamins and HDAC1 examined by Western blotting. β-Actin serves as a loading control. **e** Co-immunostaining of lamin B2 and progerin in HGPS-iPSCs derived MSCs, scar bar 10 μm. Percentages of cells exhibiting nuclear deformation and progerin expression were analyzed. Data represent mean ± S.E.; n = 4; *P < 0.05, **P < 0.01, ***P < 0.005, ****P < 0.001*p < 0.05, ** *p* < 0.01, *** *p* < 0.001, **** *p* < 0.0001; ns, not significant; unpaired two-tailed *Student’s* t-test is used for statistical analyses. **f** Immunoblotting of Ki 67 in HGPS-iPSCs and WT-iPSCs derived MSCs. Scar bar 100 μm. Data represent mean ± S.E.; n = 5; *P < 0.05, **P < 0.01, ***P < 0.005, ****P < 0.001; ns, not significant; unpaired two-tailed *Student’s* t-test is used for statistical analyses. **g** SA-β-gal staining of MSCs derived from HGPS-iPSCs and WT-iPSCs. Cellular senescence was quantified by SA-β-gal positive cell number. Data represent mean ± S.E.; n = 5, *p < 0.05, ** *p* < 0.01, *** *p* < 0.001, **** *p* < 0.0001, ns, not significant; one-way ANOVA coupled with *Tukey’s *post hoc test is used for statistical analyses. **h** Transcription of *p16*, *p21* and SASP-related inflammatory cytokine examined by qPCR. Data represent mean ± S.D.; n = 4; *p < 0.05, ** *p* < 0.01, *** *p* < 0.001, **** *p* < 0.0001, ns, not significant; one-way ANOVA coupled with *Tukey’s *post hoc test is employed for statistical analyses

We firstly generated iPSCs using dermal fibroblasts derived from a HGPS patient. The expression of progerin were confirmed in HGPS fibroblasts by Western blotting using antibodies against lamin A/C (Fig. 4d). To generate iPSC cell lines, HGSP fibroblasts at passage 5 were reprogrammed with a minicircle vector expressing 4 Yamanaka factors. Valproic acid and ascorbic acid were supplemented in the culture medium to improve the reprogramming efficiency. Among six HGPS-iPSC clones, two were chosen for further characterization. No detectable integration of vector fragments into iPSC genome was observed in both clones (Additional file 1: Fig. S3a).

Similar to H9 ESCs, both HGPS-iPSCs clones expressed pluripotency associated markers, such as *GDF3, NANOG, OCT3/4, SOX2, hTERT* and *DNMT3b* at mRNA level (Additional file 1: Fig. S3b, c). Immunostaining and immunochemistry confirmed the expression of NANOG, SSEA4, OCT3/4, TRA1-60, and AP activity in HGPS-iPSCs. In addition, reprogrammed HGPS-iPSCs can form EB (Fig. 4a). Consistent with previous reports, the transcription of lamin A, lamin C or progerin were silenced whereas lamin B1 mRNA was upregulated significantly in HGPS-iPSCs, compared with the parental fibroblasts (Additional file 1: Fig. S3d). Bisulfite sequencing revealed that the endogenous *OCT3/4* promoter region was in an open status in HGPS-iPSCs (Fig. 4b). Karyotyping and in vivo teratoma formation assay showed that HGPS-iPSCs retained the genomic integrity (44 + XY) with full differentiation potential (Additional file 1: Fig. S3e, f). HGPS cells, including fibroblasts, and smooth muscle cells (SMCs) and MSCs manifested disrupted nuclear architecture and abnormal epigenetic modifications. The absence of abnormal nuclear blabbing (Fig. 4c) and lamin A/C expression as well as the increased expression of lamin B1 and HDAC1 indicated that defects in the nucleus and epigenetics were reset in HGPS-iPSCs (Fig. 4d) and reprogramming rejuvenated the premature aging in HGPS cells.

### Recapitulation of premature aging in HGPS-iPSCs derived MSCs (HGPS-MSCs)

HGPS-iPSCs were further induced to generate MSCs using our aforementioned protocol. The differentiation of both WT-iPSCs and HGPS-iPSCs were performed in parallel. At passage 3, the MSCs differentiated from both WT-iPSCs and HGPS-iPSCs exhibited typical MSC surface markers with over 95% of cells positive for CD73, CD90, CD105 and CD44. Meanwhile, the MSCs negative markers were extremely low, with less than 0.1% of cells positive for CD11b, CD20, CD34, CD45 and HLA-DR (Additional file 1: Fig. S4a). These MSCs were able to further differentiate into adipocytes, osteocytes and chondrocytes (Additional file 1: Fig. S4b).

Further characterization of HGPS-MSCs by double staining of lamin B2 and progerin revealed the presence of progerin and nuclear abnormality. About half of the HGPS-MSCs (47.8 ± 10.3%) exhibited detectable progerin expression. Significant higher percentage of cells manifested nuclear blabbing (78.1 ± 12.7% in HGPS-MSCs Vs 21.1 ± 6.8% in WT-MSCs) (Fig. 4e). HGPS-MSCs exhibited significantly reduced proliferation (57.3 ± 5.8% in WT-MSCs Vs 7.9 ± 1.6% in HGPS-MSCs) (Fig. 4f) and remarkably increased senescence (86.7 ± 5.6% in HGPS-MSCs Vs 7.5 ± 4.3% WT-MSCs) and increased DNA damage (Additional file 1: Fig. S4c). In line with these observations and our previous findings (Liu et al. 2005), the DNA-damage checkpoint response in HGPS-MSCs was defective upon 10 Gy of γ-irradiation, as indicated by the delayed recruitment of 53BP1 (Additional file 1: Fig. S5). As expected, HGPS-MSCs manifested senescence associated secretory phenotypes (SASP) with dramatic upregulation of *IL1a, IL1b, IL6, IL8* and *PAI* (Fig. 4h). In addition, significant reduction in heterochromatin markers, such as HP1a, H3K9me3 and H3K27me3 were observed in HGPS-MSCs (Additional file 1: Fig. S6). Collectively, these results demonstrated that HGPS-MSCs derived by our protocol recapitulate the phenotypes observed in HGPS cells and can serve as a cell model for laminopathy-based premature aging.

### Correction of pathogenic mutation in HGPS cells by a CRISPR/Cas9-based double selection system

**Fig. 5:**
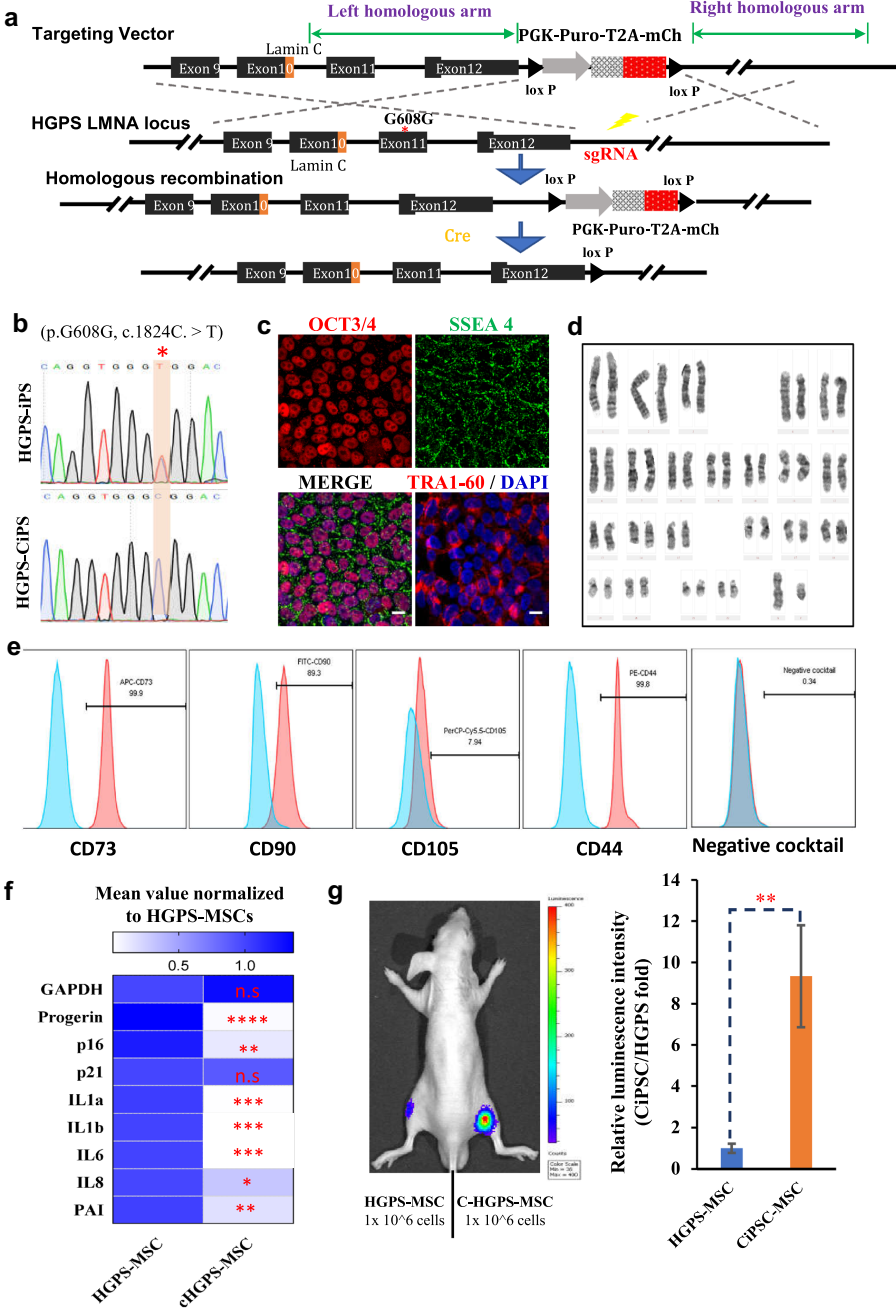
Rescue of premature senescence in mutation-rectified HGPS-MSCs. **a** Schematic illustration of the strategy to rectify *LMNA* mutation in HGPS-iPSCs by CRISPR/Cas9-mediated gene targeting. The small guide RNA targets the downstream of 3’UTR of *LMNA* gene with two homologous arms adjacent to sgRNA covering 1.5 kp upstream and downstream of genomic DNA, respectively. The double selective markers (puromycin resistant gene and fluorescent protein mCherry) are flanked by the homologous arms. The mutation corrected clones were screened and selected and confirmed by Sanger sequencing. Selection markers and other foreign DNA sequences were removed by transient expression of Flpase. **b** Sanger sequencing to confirm mutation rectification of *LMNA* gene in a HGPS-CiPSCs clone, in comparison to its parental HGPS-iPSCs. **c** Immunostaining of pluripotency markers SSEA-4, TRA60-1, NANOG and OCT3/4 (Scar bar 10 μm) in HGPS-CiPSCs. Scale bar, 10 μm. **d** Karyotyping of HGPS-CiPSCs showing normal diploid male karyotype. **e** Flow cytometry analyses of surface markers in MSCs generated from HGPS-CiPSCs. Note that more than 95% of the cells are positive for CD73, CD90, CD105 and CD44, and negative for CD45, CD34, CD11b, CD19 or HDL. **f** Heatmap of gene expressions of *progerin, p16, p21* and SASP-related genes (*IL1a, IL1b, IL6, IL8* and *PAI*), based on mean value in the MSCs derived from HGPS-iPSCs and HGPS-CiPSCs. Data represent mean ± S.D.; n = 4; * *p* < 0.05, ** *p* < 0.01, *** *p* < 0.001, **** *p* < 0.0001, ns, not significant; one-way ANOVA coupled with Tukey’s post hoc test is employed for statistical analyses. **g** Comparison of the In vivo bioluminescence between MSCs derived from mutation rectified HGPS-CiPSCs and MSCs derived from HGPS-iPSCs. Bioluminescent intensities were quantified according to the luciferase activities. Data represent mean ± S.D.; n = 3; **p < 0.01; one-way ANOVA coupled with Tukey’s post hoc test is employed for statistical analyses

As MSCs transplantation can ameliorate aging associated disorders and extend lifespan [43], it is plausible MSCs will serve as a novel therapeutic strategy for HGPS in addition to other approaches like targeting lamin A post translational process, modulating progeria or prelamin A level, activation of autophagy and reprogramming [44–46]. To fulfil this purpose and avoid potential undesired immune response, we generated genetically rectified autologous MSCs. The homologous recombination (HR) strategy was adopted to correct HGPS mutation in iPSCs derived from the patient. Earlier HR methods mainly employ a single antibiotic resistant gene cassette to enrich positive cells, which requires an additional round of screening to remove DNA fragment. To accelerate this process, we designed a double selection donor vector containing antibiotic resistant gene and fluorescent protein mCherry gene cassette flanking with a 3.5 kb and 1.7 kb homologous arm, respectively (Fig. 5a). To correct HGPS-iPSC, the guide RNA out of gene body region was designed and a specificity-enhanced Cas9 was used to minimize the undesired side-effects caused by gene editing. After nucleofection and puromycin selection, colonies were collected and expanded to screen for HGPS-iPSC clones with *LMNA* gene correction by Sanger sequencing (Fig. 5b). The mutation-corrected HGPS-iPSCs clone (HGPS-CiPSCs) was further examined for its pluripotency. As shown in Fig. 5c, the HGPS-CiPSCs were positive for SSEA4, OCT3/4 and TRA1-60. Karyotyping assay confirmed the genome integrity in HGPS-CiPSCs (Fig. 5d). We then generated MSCs from HGPS-CiPSCs. As shown in Fig. 5e, surface markers confirmed HGPS-CiPSCs-derived MSCs met the criteria of MSCs (Fig. 5e). HGPS-CiPSCs-derived MSCs showed a significant downregulation of p16 and a dramatic decline in SASP-associated genes, compared to their counterpart HGPS-MSCs (Fig. 5f). To examine the fate of these MSCs in vivo, we labelled both HGPS-MSCs-derived and HGPS-CiPSC-derived MSCs with luciferase via lentiviral infection. Equal number (10^6^ cells) of HGPS-MSCs-derived and HGPS-CiPSC- derived MSCs were inoculated into immunocompromised mice at the middle parts of left and right tibialis anterior (TA) muscle, respectively. Luminescence was measured every other day. At day 7, accelerated decay of luminescence was observed in the left leg at the engrafted site (Fig. 5g), indicating HGPS-CiPSC derived MSCs have significant survival capability in vivo. These data collectively demonstrated rejuvenation of premature senescence after mutation correction.

## Discussion

MSCs hold a great promise for a broad-range of clinical applications and regenerative medicine. Concerns over the immune response and ethical issue on the allogenic MSCs have been in debate whereas the application of autologous MSCs is constrained by the limited number of primary MSCs available. A number of methods and protocols have been developed to derive MSCs from hPSCs [19, 20, 22, 23, 47–50]. However, these methods take relatively long time and are less efficient. In this study we introduced a rapid yet robust method to generate multipotent MSCs from human pluripotent stem cells including ESCs and iPSCs. It has been reported that MSCs can be derived from pure NCCs by a two-stage induction strategy (Fukuta et al., 2014; PNAS, 2013/4). It involves either extended culture time or purification of NCCs by FACS sorting, making it difficult to obtain large scale MSCs within a short period of time. To circumvent this, we developed a new protocol in which the PSCs were initially induced to a state of neural ectoderm within 5–7 days, followed by MSC differentiation within as short as 4 days. Fully-differentiated MSCs from iPSCs or ESCs can be obtained within 2–3 weeks by our protocol, which is much shorter than current protocols through extended NCC induction that usually takes at least 5 weeks. On the other hand, our protocol provide possibility to generate MSCs in a large scale without sorting, making it quite practical for clinical applications. Importantly, these PSCs-derived MSCs exhibited standard surface markers with about 90% similarity in transcriptomic profiles and comparable differentiation potential demonstrated by tri-lineage induction, when compared with bone marrow derived primary MSCs.

HGPS is an extremely rare autosomal dominant genetic disorder in which aging associated symptoms are manifested at early stage of life. HGPS provides a useful model for aging study to reveal the underlying molecular mechanism and therapeutic strategies for physiological aging and ageing related diseases. To extend the application of our method, we generated a cell model for HGPS by reprogramming the dermal fibroblasts from a HGPS patient into iPSCs, followed by MSCs induction. One concern for clinical application of PSCs-derived MSCs is the insertional mutations by foreign DNA. To minimize the potential detrimental effects of foreign DNA during the reprogramming, we employed minicircle vector to express Yamanaka factors to avoid insertional mutations in the genome. The HGPS-MSCs derived from the progeria patient manifested irregular nuclear morphology and blabbing, increased DNA damage, defective DNA damage repair, abnormal epigenetic modification, inflammatory cytokines activation and accelerated senescence. Therefore, HGPS-MSCs can serves as a cell model for accelerated aging in vitro for mechanistic study and senolytic screening. As reprogramming silenced the *LMNA* gene in HGPS-iPSCs which is devoid of senescence, it provides an unlimited source for the production of MSCs to serve as a cellular model for HGPS.

To further extend the potential clinical application of our method, we generated *LMNA* mutation-rectified MSCs from HGPS-iPSCs. As shown in Fig. 5, *LMNA* mutation-rectified MSCs lost the production of progerin and inflammatory cytokines. Importantly, mutation-corrected MSCs were rejuvenated with a significantly improved survival when transplanted into immunocompromised mice. This provides a proof of principle in the application of our method by generating genetically rectified MSCs for autologous stem cell therapy in premature aging. It is worth to mention that the reprogramming process and MSCs induction were carried out with chemically-defined medium without animal components, making it complaint with potential clinical application.

In summary, we have developed a simple, rapid and robust method for MSCs derivation from iPSCs and ESCs. Our approach has successfully been used to establish a MSCs model for HGPS and provided a proof of principle in the rapid production of genetically rectified human autologous MSCs for potential applications in regenerative medicine.

## Material and methods

### Primary cell isolation and culture

The dermal tissues were collected from a 5-year-old male HGPS patient and a 26-year-old female normal people through skin biopsy upon the consent given by guardians and human tissue procurement under the guidance of ethical regulations in The Dongguan Women & Children Hospital, CHINA. The dermal tissues were quickly sterilized with 75% ethanol for 1–2 min, washed with 1 × PBS buffer twice, excluded extra fat and fascia, and then cut into several 1 mm^2^ pieces before seeding in T25 flask. Five milliliters of fibroblast culture medium containing DMEM/HG, 10% hUCBS, 1 mM GlutaMax, and Penicillin/ Streptomycin, was then added to the culture and the expansion of spindle-like fibroblasts was regularly monitored every 24 h. Early passages of these cells were cryopreserved with medium plus 10% DMSO. After large-scale cryopreservation, commercial Fetal Bovine Serum (Hyclone) was used to replace hUCBS for the following experiments. All the cell cultures were maintained in a humid incubator at 37 °C with 5% of CO2.

### Human umbilical cord blood serum (hUCBS) preparation

Human umbilical cord blood was collected from healthy mothers with no genetic or infectious disease history during childbirth in The Dongguan Women & Children Hospital. After fetal delivery, UCBS was harvested from the umbilical cord vein. The blood was then kept for 2–4 h at R.T. to precipitate blood cells. After 15 min of centrifugation at 15,000 RPM, the serum was carefully isolated, pooled aliquoted, sterilized with a 0.22 μm filter and transferred to -80 ℃ for long-term preservation.

### Minicircle vector mediated human iPSCs generation

The minicircle DNA mediated reprogramming was performed following previously described [51] with modifications. The minicircle vector MIP247 was a kind gift from Mark Kay & Joseph Wu (Addgene # 63,726). Early passages of human primary fibroblasts (< passage 10) were cultured as described above and 10^6 of cells were harvested and nucleofected with 15 μg plasmid using commercial kit (Lonza,Cat. No. VPD-1001) under program U-023 before seeding onto two wells of Geltrex (LifeTech) coated 6-well plate for feeder-free or autogenous feeder cells with fibroblast culture medium. Half of the medium was replenished next day, and 2 mM Valproic Acid (VPA) and 50 μg/mL ascorbic acid were added. On day 3–14, the medium was switched to N2B27 medium containing 50 μg/mL L-ascorbic acid, 50 ng/ml bFGF. Pre-iPSC clones were observed on day 7 and became larger and maturation along with reprogramming process. The medium was replaced with Essential 8 medium for feeder free or knockout serum replacement medium for feeder culture after day 15. The iPSC clones became fully-reprogramed for mechanical picking on day 25–30. At least six different clones of each sample were picked, expanded and cryopreserved. The protocol of iPSCs characterization, such as karyotyping, teratoma formation and embryonic body formation assay is same to previously described [52].

### Confirmation of minicircle DNA integration-free

Genomic DNA of iPSCs was extracted by phenol chloroform isoamyalcohol extraction and ethanol-precipitation. Minicircle specific primers with fragments homologous to genome DNA were used for PCR and agarose gel electrophoresis. The detailed information of primer sequence is list in Table 1.

**Table 1:** Summary of the sequence of oligos/primers used in this paper

Name	Oligo sequences from 5' > 3'	Name	Oligo sequences from 5' > 3'
MIP247-Inter-F1	GGCAGAAGGGCAAGAGAAG	MIP247-Inter-R1	CTCCCGCCATCTGTTGTTAG
MIP247-Inter-F2	GTGCCTGGCACCGCCATCAAT	MIP247-Inter-R2	CGAGAAGCCGCTCCACATACAGT
MIP247-Inter-F3	GACGGCTGTGGCTGGAAGTTCG	MIP247-Inter-R3	CCGCTTGGCCTCGTCGATGAA
MIP247-Inter-F4	GACAGGTGCCTCTGCGGCCAA	MIP247-Inter-R4	CGTACGCCTTGGAGCCGTACA
HGPS-GT-F1	CTAGCGAGGGCCTATTTCCCATG	pGl3.0-sgRNA-R	TGTCTCGAGGTCGAGAATTC
Sox2-qRT-F	AGCTACAGCATGATGCAGGA	Sox2-qRT-R	GGTCATGGAGTTGTACTGCA
Nanog-qRT-F	TGAACCTCAGCTACAAACAG	Nanog-qRT-R	TGGTGGTAGGAAGAGTAAAG
Rex1-qRT-F	TCGCTGAGCTGAAACAAATG	Rex1-qRT-R	CCCTTCTTGAAGGTTTACAC
Klf4-qRT-F	TCTCAAGGCACACCTGCGAA	Klf4-qRT-R	TAGTGCCTGGTCAGTTCATC
Myc-qRT-F	ACTCTGAGGAGGAACAAGAA	Myc-qRT-R	TGGAGACGTGGCACCTCTT
DNMT3B-qRT-F	AAGCTACACACAGGACTTGACAG	DNMT3B-qRT-R	AGTTCGGACAGCTGGGCTTT
TERT-qRT-F	TGTGCACCAACATCTACAAG	TERT-qRT-R	GCGTTCTTGGCTTTCAGGAT
GDF3-qRT-F	AAATGTTTGTGTTGCGGTCA	GDF3-qRT-R1	TCTGGCACAGGTGTCTTCAG
18 S-rRNA-qRT-F	GTAACCCGTTGAACCCCATT	18 S-rRNA-qRT-R	CCATCCAATCGGTAGTAGCG
GAPDH-qRT-F	CAAAGTTGTCATGGATGACC	GAPDH-qRT-R	CCATGGAGAAGGCTGGGG
LaminB1-qRT-F	GCTGCTCCTCAACTATGCTAAG	LaminB1-qRT-R	GAATTCAGTGCTGCTTCATATTCTC
LaminB2-qRT-F	AGTTGGACGAGGTCAACAAGAG	LaminB2-qRT-R	GGACTCCAGGTCCTTCACAC
LC-qRT-F	CTCAGTGACTGTGGTTGAGGA	LC-qRT-R	AGTGCAGGCTCGGCCTC
Progerin-qRT-F	CTCAGTGACTGTGGTTGAGGA	Progerin-qRT-R	TTCTGGGGGCTCTGGGCT
hLA-Exon1-qRT-F1	AATGATCGCTTGGCGGTCTAC	LA-Exon1-qRT-R1	CACCTCTTCAGACTCGGTGAT
LaminA-qRT-F1	CTGCTTCCAGGAAACTCCAC	LaminA-qRT-R1	GCCAGGGCAGAAAAGCAGAAG
OCT4-BS-F1	TTGGGATGTGTAGAGTTTGAGA	OCT4-BS-R1	TAAACCAAAACAATCCTTCTACTCC
AP2α-qRT-F1	ATTGACCTACAGTGCCCAGC	AP2α-qRT-R1	ATGCTTTGGAAATTGACGGA
HNK1-qRT-F1	CGGAAGCAGGTTTGGAGA	HNK1-qRT-R1	CGGAGACGCTCCGGACT
p75-qRT-F1	CAGGCTTTGCAGCACTCAC	p75-qRT-R1	CTGCTGCTGTTGCTGCTTCT
PAX3-qRT-F1	GCATGTTCAGCTGGGAAATC	PAX3-qRT-R1	ATGCTGTGTTTGGCCTTCTT
SOX10-qRT-F1	CTTTCTTGTGCTGCATACGG	SOX10-qRT-R1	AGCTCAGCAAGACGCTGG
ZIC1-qRT-F1	CACGTGCATGTGCTTCTTG	ZIC1-qRT-R1	GCGCTCCGAGAATTTAAAGA
SOX9-qRT-F1	AGCGAACGCACATCAAGAC	SOX9-qRT-R1	CTGTAGGCGATCTGTTGGGG
p16-qRT-F1	CGGTCGGAGGCCGATCCAG	p16-qRT-R1	GCGCCGTGGAGCAGCAGCAGCT
p21-qRT-F1	CCTGTCACTGTCTTGTACCCT	p21-qRT-R1	GCGTTTGGAGTGGTAGAAATCT
IL1α-qRT-F1	GTGCTGCTGAAGGAGATGCCTGA	IL1α-qRT-R1	CCCCTGCCAAGCACACCCAGTA
IL1β-qRT-F1	TGCACGCTCCGGGACTCACA	IL1β-qRT-R1	CATGGAGAACACCACTTGTTGCTCC
IL6-qRT-F1	CCAGGAGCCCAGCTATGAAC	IL6-qRT-R1	CCCAGGGAGAAGGCAACTG
IL8-qRT-F1	GAGTGGACCACACTGCGCCA	IL8-qRT-R1	TCCACAACCCTCTGCACCCAGT
PAI-1-qRT-F1	CCTGGCCTCAGACTTCGGGGT	PAI-1-qRT-R1	GGGGCCATGCCCTTGTCATCAAT

### Bisulfite genomic sequencing

Bisulfite treatment was carried out using the DNA Bisulfite Conversion Kit (TIANGEN BIOTECH). Converted DNA was used as template for PCR to analyze DNA methylation state of endogenous *OCT4* promoter region using primers in oligo tables. PCR was performed using PrimeSTAR (Takara) and PCR products were purified and cloned into pJET1.2 vector. Sequences of 10 random picked bacterial clones were analyzed. Each column of circles for a given amplicon represents the methylation status of CpG dinucleotides in one clone for that region. Open circles are unmethylated CpGs and closed circles methylated ones. The left numbers of each column indicate CpG localization relative to the transcriptional start site. The detailed information of primer sequence is list in Table 1.

### Reverse-Transcription PCR and quantitative PCR

RNA was extracted from culture cells by Trizol (Sigma) and 1 μg RNA was used for cDNA synthesis. For reverse PCR, cDNAs were diluted to 200-fold as template and high-fidelity DNA polymerase was used. For qPCR, SYBR Green PCR Kit (Applied Biosystems) was used with cDNAs diluted 50-fold as template and PCR was carried out at standard thermal cycling conditions (95 ℃ for 20 s; 40 cycles 95 ℃ for 20 s; 60℃ for 30 s. For melting curve, 95 ℃ for 15 s; 60℃ for 1 min; 95 ℃ for 15 s), three different repeats were performed and _△△_CT values were calculated for statistical analysis. The detailed information of primer sequence is list in Table 1.

### Immunostaining

The cells were blocked with blocking buffer (PBST containing 5% FBS and 5% BSA) for 1 h after fix and permeabilization before incubated with primary antibodies at 4 °C overnight. After washing with PBST, the cells were incubated in secondary fluorescent antibodies for 1 h and counterstained with DAPI (Life Technology). Images were acquired with the confocal microscope (Carl Zeiss LSM800) and were processed with ZEN Blue software. For DNA damage response assay, cells were exposed to 10 Gy γ-irradiation. The detailed information of antibodies used are given in Table 2.

**Table 2:** Summary of antibodies used in this paper

Antibody	Species	Vendor	Cat.NO		WB dilution		IF dilution
HP1 alpha	Rb	Abcam	ab109028		1/3000		1/250
H3K9me3	Rb	Abcam	ab176916		N/A		1/200
H3K27me3	Rb	Abcam	ab6002		N/A		1/200
SOX10	Rb	Abcam	ab155279		1/1500		1/100
Nestin	Rb	Beyotime	AF2215		N/A		1/50
LMNB2	Rb	Beyotime	AF0219		1/1000–1/5000		1/200
Lamin B1	Rb	Beyotime	AF1408		1/1500		N/A
Lamin B1	Rb	Proteintech	12987-1-AP		1/2000		1/50
Lamin A/C	Rb	Proteintech	10298-1-AP		1/1000		1/100
Nanog	Ms	Santa Cruz	sc-374001		N/A		1/50
Oct-3/4	Ms	Santa Cruz	sc-5279		N/A		1/50
SSEA-4	Ms	Santa Cruz	sc-21704		N/A		1/50
TRA-1–60	Ms	Santa Cruz	sc-21705		N/A		1/50
Progerin	Ms	Sigma	SAB4200272		1/3000		1/200
γH2A.X	Ms	Millipore	05-636		N/A		1/100
53BP1	Rb	Santa Cruz	sc-22760		N/A		1/50

### Cell lysis and Western blot

The cells were lysed in RIPA buffer containing protease inhibitor (Roche) and incubated on ice for 30 min. Cell lysate was then centrifuged 1,3000 rpm at 4 °C and the soluble proteins were boiled with loading dye before subjected to electrophoresis in 8% SDS-PAGE gel for separation and western blotting.

### MSCs generation via temporal neuralized ectoderm induction of hPSCs

hPSCs is routinely cultured in mTeSR1 or Essential 8 media with splitting at 1:4–1:6 every 3 days. For neuralized ectoderm differentiation, 1 × 10^5 of signalized hPSCs were seeded on Matrigel coated 6-well plate with 10 μM Y27632. The medium was discarded, washed with DMEM:F12 twice and changed to neural ectoderm induction medium, consisting of 50% Neurobasal, 50% DMEM/F12 medium, 1% N2 and 2% B27 and 3–6 μM CHIR-99021 and 10 μM SB-431542. Replenish the medium daily for 5 days until the cells exhibited neural-like morphology, spread out and separated from each other. Signalized the cells and re-seeding them on Matrigel-coated plate with MSC culture medium, consisting of alpha- MEM basal medium, 10% FBS, 1X Glutamax solution, 5 ng/ml bFGF, 5 ng/ml EGF and 1X Penicillin–Streptomycin. After additional 5–7 days of induction, fibroblast-like morphology is observed. Split the cell at low ratio, such as 1:2–1:3 at early passages with TrypLE Express. Flow cytometry analysis to characterize MSC surface markers are usually performed at passage 3–5.

### Flow cytometry analysis of MSCs surface markers

When MSCs reached 80–90% confluency, cells were washed with 1 × PBS, dissected to single cells, and finally resuspended in 1.2 ml of 1 × PBS containing 3% FBS. Cells were aliquoted equally to six different tubes and stained with fluorescent antibodies (BD 562,245) for 30 min protected from light. Cells were washed twice with 1 × PBS and resuspended with 150 µl 1 × PBS containing 3% FBS and transferred to Flow Cytometry Tubes for BD FACSAria SORP analysis. Data were analyzed with FlowJo 10.7.

### Tri-lineage differentiation of MSCs

#### Adipogenesis

For adipocyte differentiation, cells growing to 90–100% confluency were washed with 1X PBS for three times before switched to adipocyte induction medium, consisting of HG-DMEM basal medium supplemented with 10% FBS, 0.5 mM 3-isobutyl-1-methylxanthine (IBMX), 100 µM Indomethacin, 1 µM Dexamethasone, 10 µg/ml Insulin and 1X Penicillin–Streptomycin solution, with medium change every 3 days. After 21 days of induction, cells were fixed in 4% paraformaldehyde and stained with Oil Red O solution.

#### Osteogenesis

For osteoblast differentiation, cells growing to 90–100% confluency were washed with 1X PBS for three times before switched to osteoblast induction medium, consisting of HG-DMEM basal medium supplemented with 10% FBS, 50 μg/ml L-Ascorbic acid, 0.1 µM Dexamethasone, 10 mM β-glycerophosphate, 1 mM Sodium pyruvate and 1X Penicillin–Streptomycin solution, with medium change every 3 days. After 21 days of induction, cells were fixed in 4% paraformaldehyde and stained with Alizarin Red S solution.

#### Chondrogenesis

For chondrocyte differentiation, cells growing to 90–100% confluency were washed with 1X PBS for three times before switched to chondrocyte induction medium, consisting of LG-DMEM basal medium supplemented with 10% FBS, 50 μg/ml L-Ascorbic acid, 0.1 µM Dexamethasone, 10 ng/ml TGFβ3, 1 mM Sodium pyruvate, 1X Insulin-Transferrin-Selenium solution and 1X Penicillin–Streptomycin solution, with medium change every 3 days. After 21 days of induction, cells were fixed in 4% paraformaldehyde and stained with Alcian blue solution.

### CRISPR/Cas9-based Double selection strategy or mutation correction

The gene-editing associated plasmids were ordered from Addgene, eSpCas9 (1.1) (#71814) and pGL3.0-sgRNA (#51133). To exclude the possibility of gene editing caused side-effects, small guide RNA (sgRNA) was designed to target noncoding regions, outside of human LMNA genome locus. sgRNA was designed with Benchling (https://benchling.com) according to Sp-Cas9 PAM (NGG) module and on/off target scores. Oligos were synthesized from IDT and annealed into pGL3.0-sgRNA vector. For the donor vector, left and right homologous arms were amplified from wild-type H9 genome DNA with 3.5 kb and 1.2 kb, respectively, and assembled with lox P-PGK-Puro-T2A-mCherry-lox P fragment via Gibson assembly. Plasmid sequence was confirmed with sanger sequencing. Total 30 µg of plasmids Cas9: sgRNA: donor vector (4:1:5) were delivered to iPSCs with nucleofection using program B-16 (Amaxa nucleofector). The cells were selected using 500 ng/ml puromycin 48 h post nucleofection for a week and seeded at low-density to Matrigel-coated dish for single cell colonies picking. Puro-mCherry double labelling clones were picked, transferred to 48-well plate, and expanded for genome typing. HGPS mutant region was amplified by PCR using Taq DNA Polymerase (TAKARA) and mutation correction were checked with sanger sequencing. The corrected clones only exhibited a unique C in LMNA c.1824C. > T but not C/T signal. The corrected single clone (HGPS-CiPSC) was further transduced with pCAG-Cre-2A-GFP to remove PGK-Puro-T2A-mCherry double labelling cassette. The mCherry negative cells were collected as desired HGPS-CiPSC with FACS sorting.

### Senescence β-galactosidase staining

SA-b-gal staining was performed using a commercial kit (Beyotime Biotechnology) according to instruction manual. In brief, cells were fixed in fixative at RT for 5 min. After fixation, cells were stained with freshly prepared staining solution at 37 °C overnight. Images were taken and the percentage of senescent cells were analysed by ImageJ for quantitative analysis.

### In vivo bioluminescence of MSCs

Lentivirus vector containing human PGK promoter driven luciferase gene cassette was packaged by co-transfection psPAX2 and pMD2.G into HEK293T and the virus was concentrated by ultracentrifuge 35,000 RPM for 3 h. MSCs was infected with the virus and were selected with 10 ng/ml Blasticidin S after 48 h incubation. The stable MSC-luciferase cells were expanded and harvested for in vivo injection. About 1 × 10^6 cells were re-suspended in 100 µl PBS and injected into midportion of the tibialis anterior (TA) muscle of nude mouse. Mouse were anaesthetized and also treated with D-luciferin (150 µg/g ratio) every other day. Photon emission was detected and measured using IVIS lumina system. Bioluminescence images were acquired and quantified for statistical analysis.

## Supplementary Information

**Additional file 1.** Supplementary figures.

## Data Availability

The datasets generated and/or analyzed during the current study have been submitted to Sequence Read Archive (SRA): PRJNA785674. They are also available upon publishment of this paper or from the corresponding author on reasonable request.

## Abbreviations

MSCs: Mesenchymal stem cells
HGPS: Hutchinson-Gilford progeria syndrome
iPSCs: Induced pluripotent stem cells
ESCs: Embryonic stem cells
HSCs: Hematopoietic stem cell
SCNT: Somatic cell nuclear transfer
hUCBS: Human umbilical cord blood serum
BM: Bone marrow
sgRNA: Small guide RNA
