# Supplementary material for: Rapid and robust derivation of mesenchymal stem cells from human pluripotent stem cells via temporal induction of neuralized ectoderm
Source: Cell Biosci. 2022 Mar 15;12:31. doi: 10.1186/s13578-022-00753-2 (PMC8922747; doi:10.1186/s13578-022-00753-2)
Supplement: Supplementary file 1 — Additional file 1. Supplementary figures. [file 13578_2022_753_MOESM1_ESM.docx]

**Supplementary Information**

**Rapid and robust derivation of mesenchymal stem cells from human pluripotent stem cells via temporal neuralized ectoderm induction**

Wei Jin, Yi He, Tuo Li, Fei Long, Xin Qin, Yuan Yuan, Ge Gao, Hosen Md Shakhawat, Guoxiang Jin, Zhongjun Zhou

**
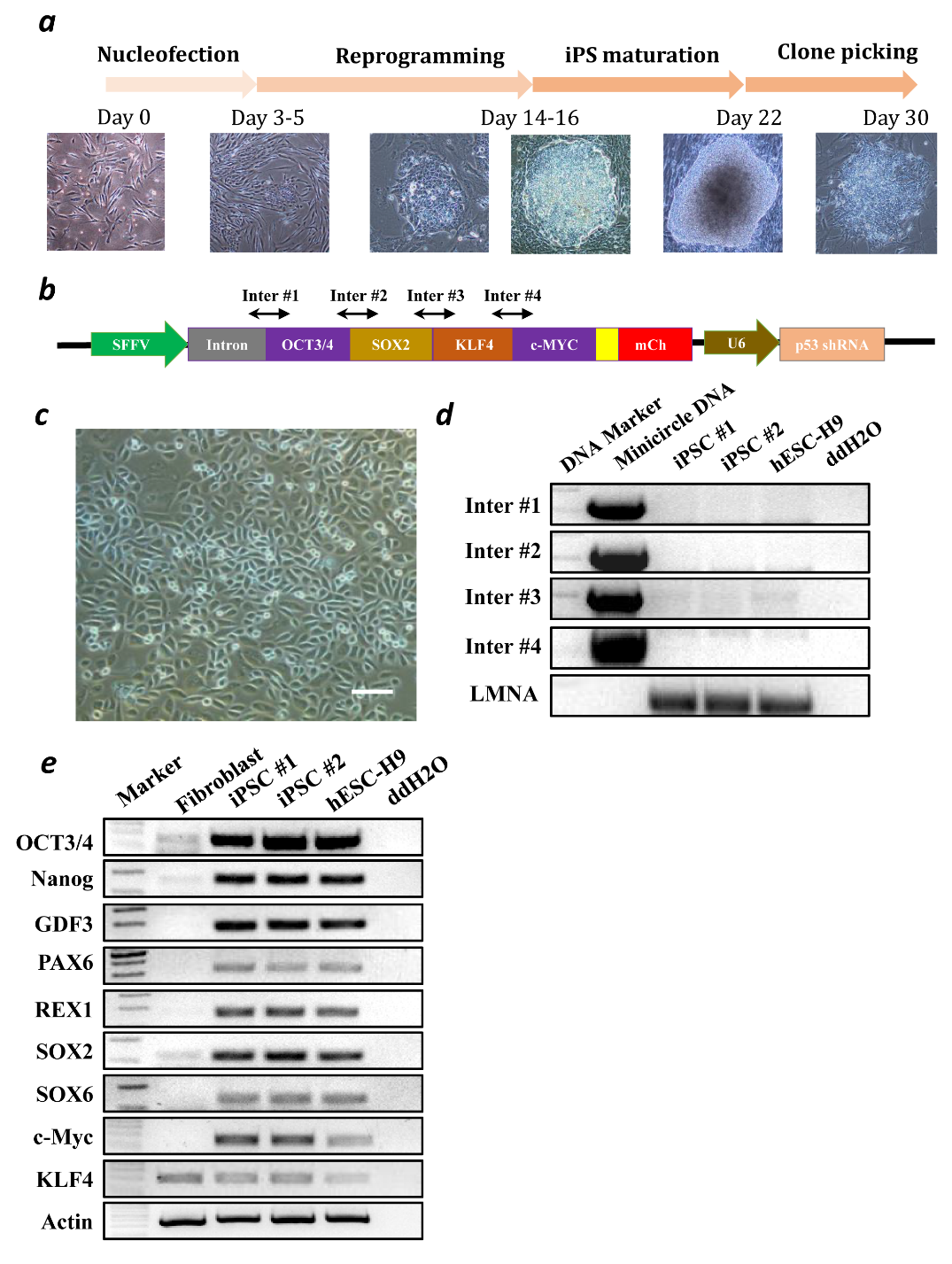
**

**Supplementary Figure 1*.* Generation and characterization of human iPSCs with an integration-free minicircle vector.**

**a.** Schematic illustration of iPSCs generation timeline and cellular morphology changes along reprogramming.

**b**. Diagram of four-in-one minicircle vector used for non-integrating reprogramming. Yamanaka factors (OCT3/4, KLF4, c-Myc, SOX2) and fluorescent protein mCherry are assembled within one gene cassette, linked with 2A peptide under SSFV promoter; U6 promoter-driven *TP53* shRNA used to increase the efficiency of iPSCs generation.

**c**. Human urinary primary epithelial cells expanded for 10 days. Scar bar 100μm.

**d**. PCR to examine the potential insertion of the reprogramming construction in 4 iPSC clones. Four pairs of PCR primer set to amply Inter #1, Inter #2, Inter #3, and Inter #4 regions are shown in panel ***a.*** Genome *LMNA* locus serves as endogenous control. Minicircle DNA serves as positive control whereas human embryonic stem cell H9 serves as negative control.

**e.** Reverse transcription PCR (RT-PCR) to detect the expression of pluripotency genes including *OCT3/4, NANOG, SOX2, SOX6, Klf4, REX1, GDF3, c-Myc, PAX 6* in iPSC and parental fibroblasts. Actin serves as a control.

**
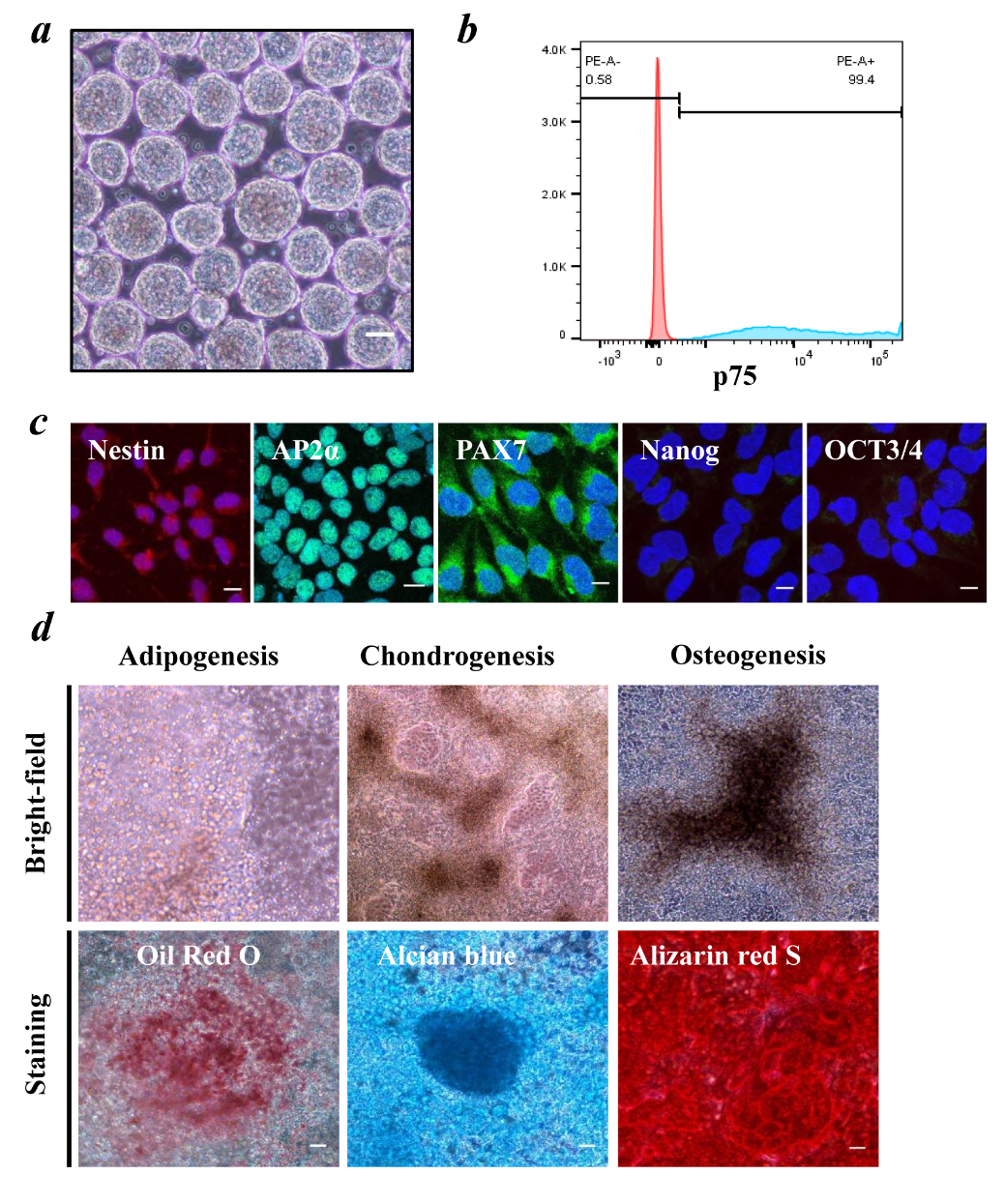
**

**Supplementary Figure 2. Generation of MSCs from pluripotent stem cells by a simple two-step induction via neuralized ectoderm as a differentiation intermediate.**

**a**. Sphere formation of neural ectoderm cells after 5 days induction of human iPSCs. Scar bar 100 μm.

**b**. Flow cytometry analysis of the neural ectoderm intermediate, showing the vast majority of cells are p75 positive.

**c**. Immunostaining of the neural ectoderm intermediate with HNK1, AP2a, PAX7, Nestin, NANOG and OCT3/4. Scar bar 10 μm.

**d**. Differentiation of neural ectoderm intermediate toward adipogenesis, chondrogenesis and osteogenesis. Adipocyte, chondrocyte, and osteoblast are detected by the staining of Oil red O, Alizarin red S and Alcian blue, respectively. Scar bar 100 μm.


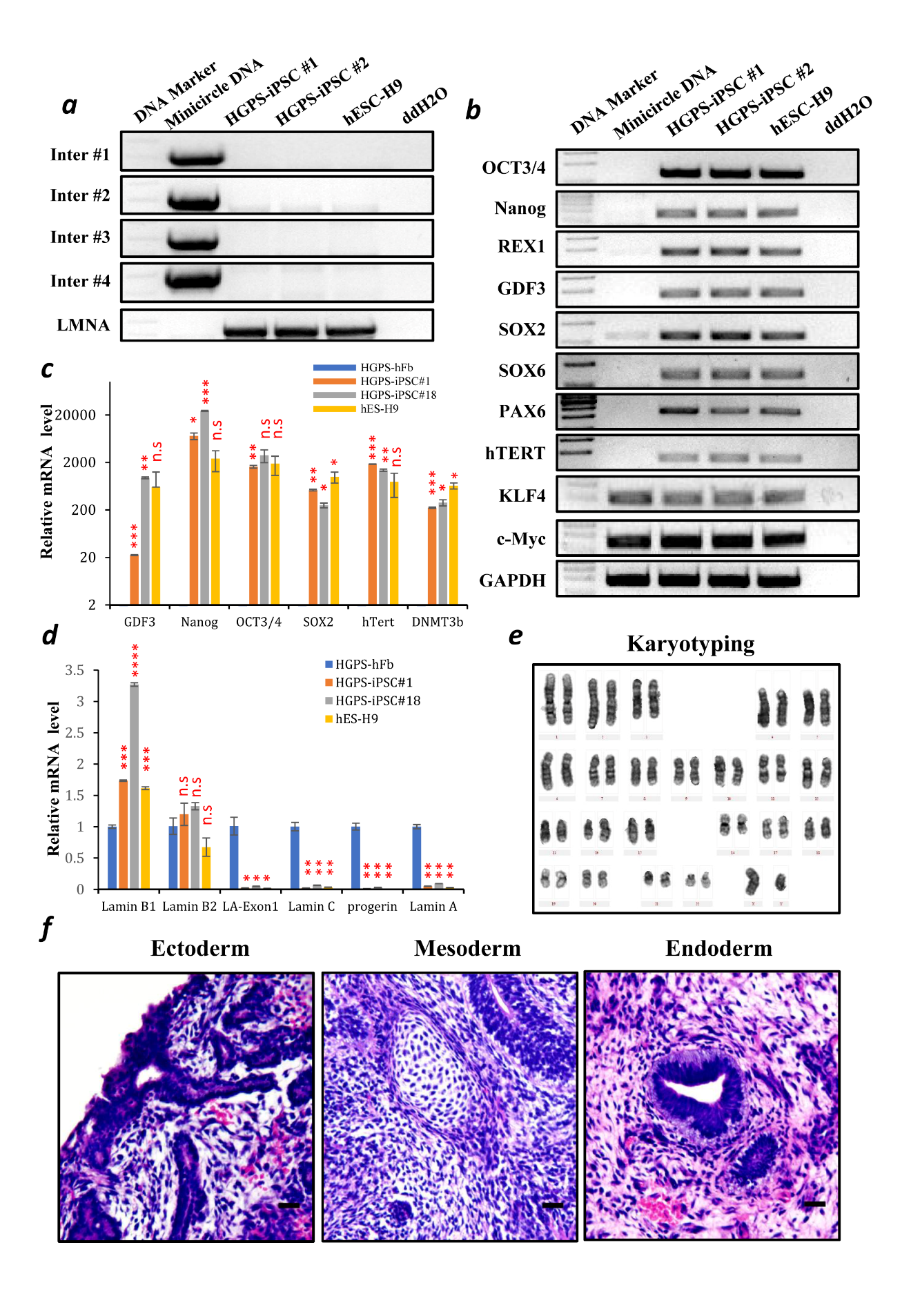


**Supplementary Figure 3*.*  Characterization of HGPS patient specific iPSCs.**

**a**. PCR to examine potential insertion of reprogramming construct in two HGPS-iPSCs clones using 4 pairs of primers to amplify the designated region indicated in Supplementary Figure 1a. Genome *LMNA* locus serves as endogenous control. Minicircle DNA serves as positive control and genomic DNA from human embryonic stem cells H9 serves as negative control.

**b.** Reverse transcription PCR (RT-PCR) to detect pluripotency related genes including *OCT3/4, Nanog, SOX2, SOX6, Klf4, REX1, GDF3, c-Myc*, and *PAX 6* in two HGPS-iPSCs clones and parental fibroblasts. Actin serves as a control.

**c**. qPCR analyses of pluripotency related genes *OCT3/4, NANOG, SOX2, REX1, GDF3, hTERT* and *DNMT3b* in two HGPS-iPSCs clones. Gene expression was normalized to the endogenous *GAPDH*. Relative mRNA levels were plotted against to that in parental fibroblasts. H9 ESCs serve as the positive control. Data represent mean ± S.D.; n=3.

**d**. qPCR analyses of nuclear lamins including lamin B1, lamin B2, lamin A, lamin C and progeirn in HGPS-iPSCs. Gene expression was normalized to the endogenous *GAPDH*. Relative mRNA levels were plotted against to that in parental fibroblasts. Human ESCs H9 serve as a positive control. Data represent mean ± S.D.; n=3.

**e**. Karyotyping of HGPS-iPSCs showing normal diploid female karyotype.

**f**. Teratoma formation of HGPS-iPSCs in the testis of NOD/SCID mice. H&E staining of teratoma paraffin sections to reveal three germ layers. Scar bar 100 μm.

**
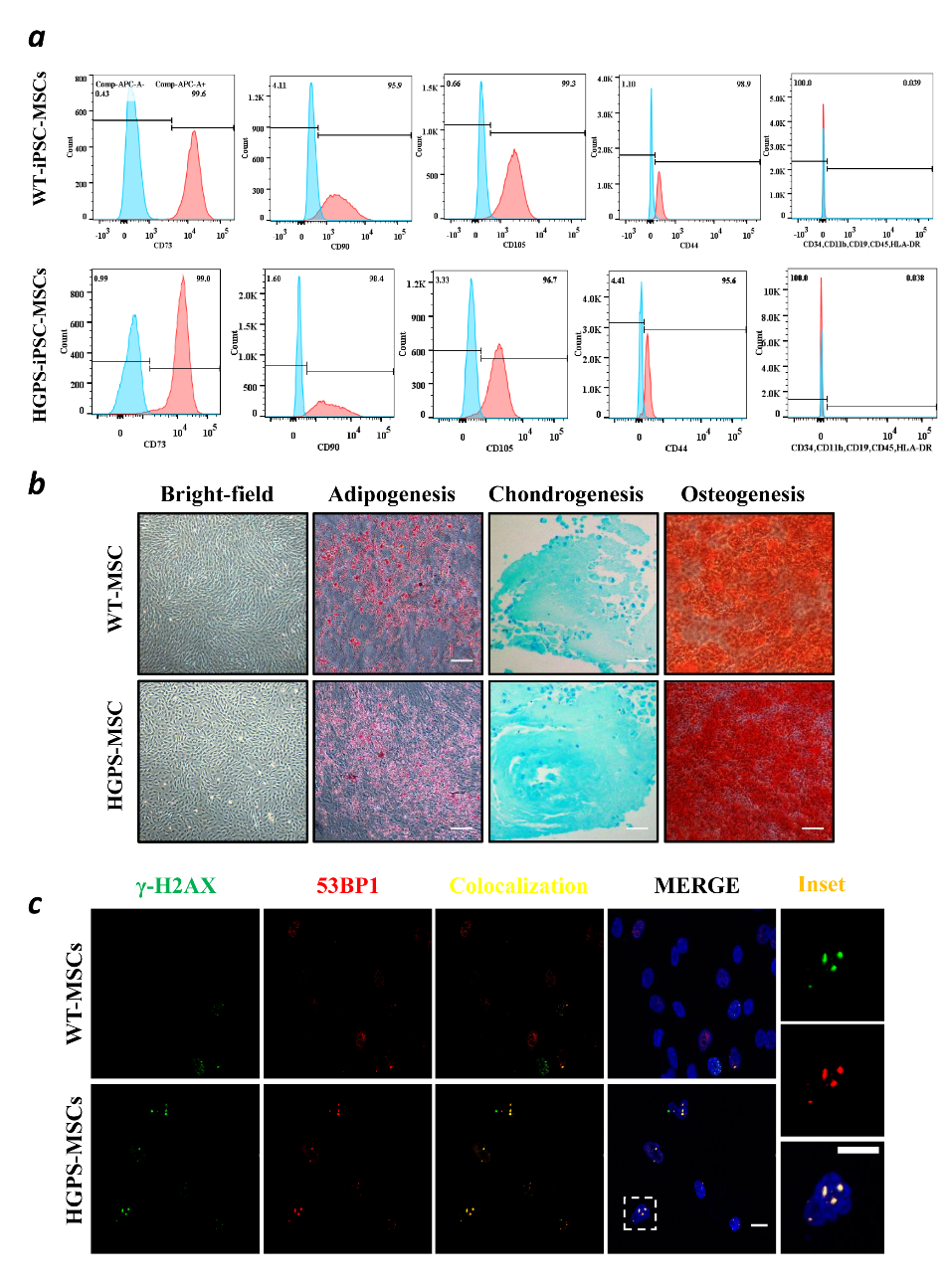
**

**Supplementary Figure 4. Generation and characterization of HGPS iPSCs derived MSCs.**

**a**. Flow cytometry analyses of typical positive (CD73, CD90, CD105 and CD44) and negative (CD45, CD34, CD11b, CD19 nor HDL-DR) MSCs surface markers in WT-MSCs and HGPS-MSCs.

**b**. Morphology and multiple differentiation potential of WT-MSCs and HGPS-MSCs. Adipocytes, chondrocytes, and osteoblasts detected by staining of Oil red O, Alizarin red S and Alcian blue, respectively. Scar bar 100 μm.

**c**. Co-immunostaining of γ-H2AX and 53BP1 in WT-MSCs and HGPS-MSCs at passage 15. The white box in HGPS-MSCs is enlarged in the right panels. Scar bar 10 μm.

**
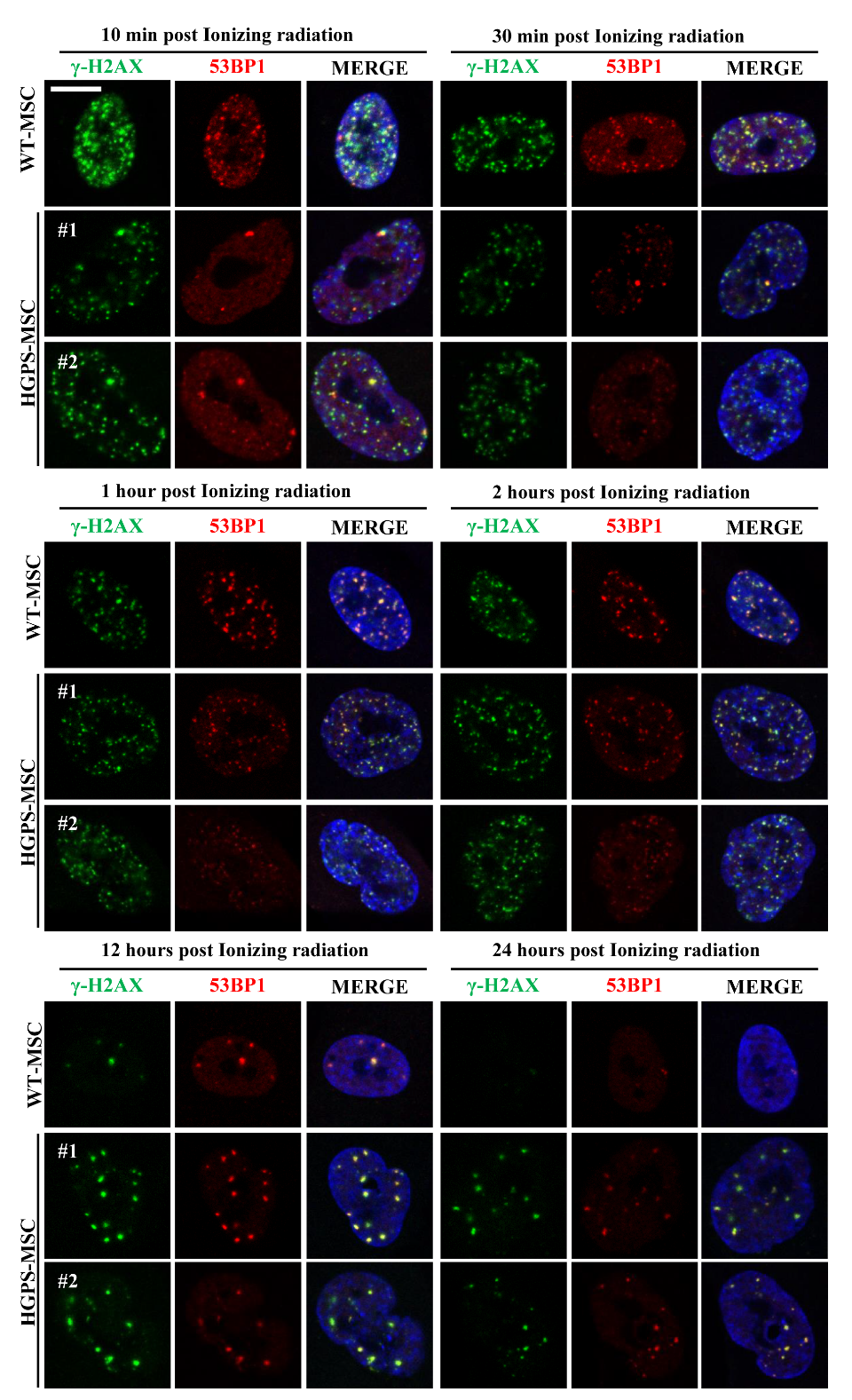
**

**Supplementary Figure 5*.*  The kinetics of DNA damage checkpoint response in WT-MSCs and HGPS-MSCs.**

Immunofluorescence analyses of γ-H2AX and 53BP1 in WT-MSCs and HGPS-MSCs at different time points upon 10 Gy of γ-irradiation. Scale bars, 10 μm.

**
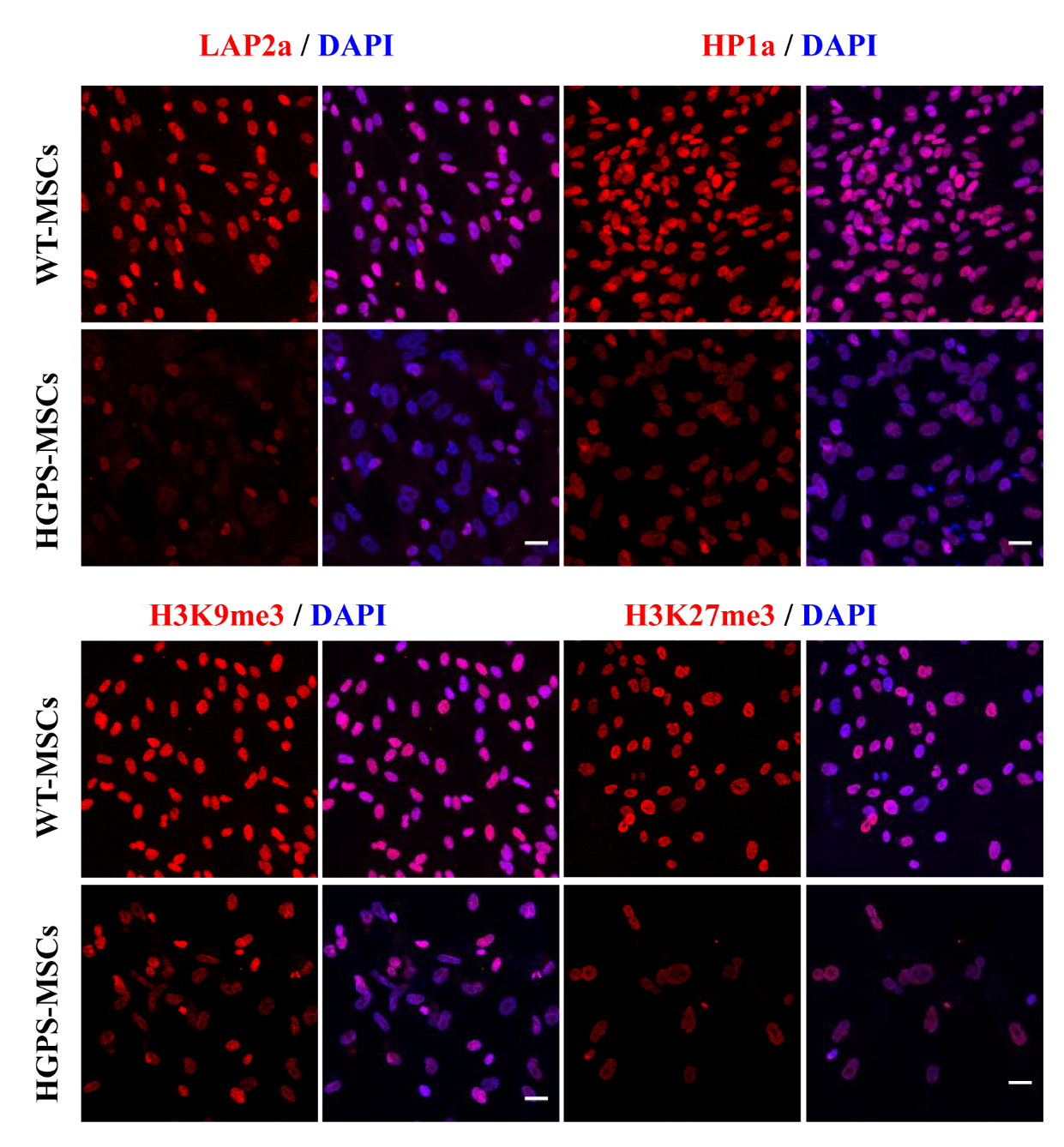
Supplementary Figure 6*.*  Epigenetic alternations in HGPS MSCs.**

Immunofluorescence analyses of LAP2a, HP1a, H3K9me3 and H3K27me3 in WT-MSCs and HGPS-MSCs. Scale bars, 10 μm.
